# How language production shapes language form and comprehension

**DOI:** 10.3389/fpsyg.2013.00226

**Published:** 2013-04-26

**Authors:** Maryellen C. MacDonald

**Affiliations:** Department of Psychology, University of Wisconsin-MadisonMadison, WI, USA

**Keywords:** language acquisition, motor control, language production, serial order, language comprehension, syntax, language typology, working memory

## Abstract

Language production processes can provide insight into how language comprehension works and language typology—why languages tend to have certain characteristics more often than others. Drawing on work in memory retrieval, motor planning, and serial order in action planning, the Production-Distribution-Comprehension (PDC) account links work in the fields of language production, typology, and comprehension: (1) faced with substantial computational burdens of planning and producing utterances, language producers implicitly follow three biases in utterance planning that promote word order choices that reduce these burdens, thereby improving production fluency. (2) These choices, repeated over many utterances and individuals, shape the distributions of utterance forms in language. The claim that language form stems in large degree from producers' attempts to mitigate utterance planning difficulty is contrasted with alternative accounts in which form is driven by language use more broadly, language acquisition processes, or producers' attempts to create language forms that are easily understood by comprehenders. (3) Language perceivers implicitly learn the statistical regularities in their linguistic input, and they use this prior experience to guide comprehension of subsequent language. In particular, they learn to predict the sequential structure of linguistic signals, based on the statistics of previously-encountered input. Thus, key aspects of comprehension behavior are tied to lexico-syntactic statistics in the language, which in turn derive from utterance planning biases promoting production of comparatively easy utterance forms over more difficult ones. This approach contrasts with classic theories in which comprehension behaviors are attributed to innate design features of the language comprehension system and associated working memory. The PDC instead links basic features of comprehension to a different source: production processes that shape language form.

## Introduction

Humans are capable of a remarkable number of highly complex behaviors—we plan ahead, remember the past, reason, infer, and invent. The origins of intelligent behavior are at the core of classic debates in cognitive science concerning the contributions of innate capacities and experience in the development of thought, perception, and action. For example, the fact that perception of motion in cardinal directions (vertical, horizontal) is superior to that in oblique directions has been attributed to the greater number of cells in visual cortex devoted to processing cardinal motion directions than oblique ones (Rokem and Silver, 2009), and this result in turn is thought to arise from visual experience: There are more motion events in the world in cardinal directions than in oblique ones (Dakin et al., 2005). Similarly, experience-based accounts of face perception hold that face recognition behavior diverges from object recognition because perceivers' visual experience with faces differs in critical ways from their experience with objects (Tarr and Gauthier, 2000). While such accounts don't deny innate factors in perception, they are notable in ascribing a central role for experience in development and in adult performance.

The statistical properties of the input have a similarly crucial role in some accounts of language use, including the role of linguistic experience in acquisition (Hart and Risley, 1995) and in adult comprehension processes (MacDonald et al., 1994). However, the nature of the argument is critically different in vision and in language. Visual experience reflects the nature of the physical world: Vision scientists do not need to explain why gravity creates many experiences of downward motion, and no one expects face perception researchers to explain why faces have particular shapes. In language, however, the input to the perceiver is itself the consequence of language behavior—it is the utterances produced by other language users, who have their own cognitive systems presumably shaped by their own experiences. This situation lends potential circularity to experience-based accounts of language (Frazier, 1995), requiring solutions for two unknowns at once: as in vision, language researchers must develop an account of the effects of experience on perception, but unlike in vision, language researchers must also consider why the experience—the language—has the character it does. This difficult task is compounded by the fact that the psycholinguists who study language use are typically not the same people as the linguists who study the nature of language form, so that there is a gulf between linguistic theories of the nature of language and psycholinguists' accounts of the effects of experience with language patterns.

This article is a step toward bridging this divide, offering insight into both the origin of language form and also the effect of experience with these forms. The Production-Distribution-Comprehension (PDC) account, first sketched in MacDonald (1999) and elaborated in work described here, holds that the memory and planning demands of language production strongly affect the form of producers' utterances. Constraints imposed by the production process have two important consequences. First, they contribute to understanding regularities in linguistic form: why languages exhibit particular properties, with different frequencies across languages. Second, they determine many aspects of language comprehension. The claim is not that all aspects of language form and comprehension can be traced to the computational demands of language production, but rather that production's impact in these areas is so pervasive that understanding production becomes essential to explaining why language is the way it is, and why language comprehension works the way it does.

In this article I describe the Production, Distribution, and Comprehension components of the PDC in that order, focusing particularly on lexico-syntactic phenomena. The section entitled *The First Step in the PDC: Production Difficulty and its Amelioration* reviews the memory and control demands of language production, producers' attempts to mitigate them, and the patterns of word order, sentence form, and lexical-sentence pairings that result. Findings in motor control, memory retrieval, and short term maintenance suggest that many properties of language production that affect utterance form also arise in action and motor planning more generally. Next, the section entitled *Distributional Regularities and Language Typology* considers the effects of language production on language form and views the potential contributions of the PDC in the context of other accounts of why languages have some properties more than others. Finally, *Comprehension Consequences in the PDC* addresses comprehension, showing that the PDC provides a different framework for thinking about sentence comprehension and offers a different explanation of some classic results.

## The first step in the PDC: production difficulty and its amelioration

Language production is a highly complex motor behavior, requiring the translation of conceptual information into an intricate sequence of motor commands to allow speaking, signing, writing, or typing. Although “production difficulty” and “motor control” might suggest a discussion of articulation, here we consider difficulty arising in the development of the plan for the utterance, well ahead of articulation^1^. Lashley (1951) considered the development and organization of plans for output sequences as “both the most important and also the most neglected problem in cerebral physiology” (p. 114). He argued that complex sequential actions such as speaking must be guided by a plan that is developed before execution, a view that continues to pervade research in motor behavior, including language production. The construction of motor plans is a cognitively demanding activity; developing the utterance plan can be more demanding than speaking itself (Kemper et al., 2011). The significant computational difficulty of constructing and maintaining an utterance plan is a key component of the PDC, and so we consider these planning operations in some detail.

### Development and care of the utterance plan

Language planning shares features of both high-level non-linguistic action planning and more fine grained motor control. In high-level action plans, some elements have only loosely constrained sequences. In making coffee, an example extensively discussed in research on action planning and control (Cooper and Shallice, 2000; Botvinick and Plaut, 2004), the coffee, cream, and sugar can go into the cup in any order. Similarly, in some (though by no means all) aspects of language planning, some elements may be ordered in several ways, as in *Jane bought a hammer and some batteries at the hardware store*, vs. *At the hardware store, Jane bought some batteries and a hammer.* Other aspects of action/motor plans are far more constrained—one must move the hand to the coffee cup before grasping it, and in the case of language planning, there are language-specific constraints limiting the range of permissible word orders, for example excluding *hardware at store the*. Thus language producers have word order options in some cases but not others, and when there are options, producers must very rapidly settle on one form and inhibit others from interfering, so as not to make speech error blends of alternative forms such as *some hammer and a batteries*. This behavior is an example of a winner-take-all process, and winner-take-all neural mechanisms form an important part of accounts and computational models of both language production (Hartley and Houghton, 1996; Dell et al., 1997) and non-linguistic motor behavior, including visual search (Ferrera, 2000) and the “syntax” of birdsong (Jin, 2009). This winner-take-all property of language production is critical in accounts of how producers activate the correct serial order of elements in articulation (Hartley and Houghton, 1996), and it provides our first example of how properties of motor planning affect distributional patterns in the language, in that this property affects the incidence of speech errors.

The developing utterance plan must be maintained in an executable state as it is being developed. The plan is effectively “the memory for what is to come” (Rosenbaum et al., 2007, p. 528), with all the maintenance burdens of other short-term memories. Indeed, verbal working memory studies offer important insights into some of the memory demands of language production. In both serial recall tasks (in which unrelated words are recalled in the same order they were presented) and language production tasks (such as describing pictures), elements in the utterance plan tend to interfere with one another, affecting the fluency of speech. For example, phonological overlap among elements in the utterance plan increases the difficulty in both production and memory tasks (Acheson and MacDonald, 2009), and semantic overlap between words increases errors in language production (Smith and Wheeldon, 2004) and memory tasks (Tse et al., 2011). Conversely, production of the correct serial order of elements is improved by increased linguistic frequency or coarticulatory experience, both for memory tasks (Woodward et al., 2008) and language production (Dell et al., 1997). Thus, production planning has inherent working memory demands, with consequent interference and other pitfalls well known to memory researchers.

Because planning precedes execution, a key question in language production concerns the degree of advance planning before execution begins. Language production is said to be *incremental*, meaning that partial planning, execution, and subsequent planning are interleaved. The scope of advance planning varies in different circumstances and is at least partially under the producer's strategic control (Ferreira and Swets, 2002). Again, production behavior is shaped by learned implicit strategies that maximize fluency, as the scope of planning strikes a balance between competing demands. On the one hand, initiating execution before much planning is complete allows producers to begin speaking earlier, avoiding long pauses and retaining the floor in a conversation. Early execution also avoids the memory burden of maintaining and executing a large plan, as more complex plans require more time to initiate execution, both in speech (Ferreira, 1991) and in non-linguistic motor behaviors (Rosenbaum et al., 2007). However, interleaving planning and execution has the occasional negative consequence of the producer finishing the executable portion of the plan before the next portion is ready. Rather than letting everything grind to a halt, speakers in this situation attempt to gain extra planning time by lengthening words or adding optional words and pauses, yielding utterances such as “Have you seen theee … um …?” (Fox Tree and Clark, 1997; Ferreira and Dell, 2000).

Beyond juggling planning and executing, language producers must also keep track of where they are in the plan as it is being executed. Tracking the state of progress through the plan is critical for avoiding repetitions, omissions and other sequencing errors, but it comes at a cost, in that tracking plan progress itself carries substantial additional attention or maintenance burdens (Botvinick and Plaut, 2004). At the same time, the memory for what has been uttered cannot remain too strong, because recently-executed actions can interfere with upcoming ones, leading to perseverations and other errors (Tydgat et al., 2012). The speaker must therefore balance the various subtasks in utterance planning in order to “activate the present, deactivate the past, and prepare to activate the future” (Dell et al., 1997, p. 123; a non-linguistic example is Deco and Rolls, 2005). An efficient allocation of attention to past, present, and future is learned over time: Fluent adult speech reflects a bias toward the future, with comparatively more anticipation errors (elements of the upcoming plan incorrectly influencing the current execution) than perseverations of previously-uttered elements (Dell et al., 1997). By contrast, young children, who are less experienced speakers, produce more errors overall and a relatively higher proportion of perseverations (Stemberger, 1989). The impact of these phenomena is threefold. First, they illuminate the demands of language planning, which include developing the plan, maintaining it, monitoring the state of execution, and shifting attentional focus as the plan is executed over time. Second, they illustrate how speakers learn implicit strategies to mitigate production difficulty, in this case learning to allocate more attention to the upcoming plan as they become more fluent, and learning to favor early execution and incremental planning, with delaying tactics and additional damage control if the plan runs out. And third, production-related learning affects the distribution of utterance forms that people produce, in this case the rate and distribution of speech errors and pauses in utterances. The intersection of these last two points—that the computational demands of language production can be mitigated, but with consequences for utterance form—will reappear below as a force in the distribution of syntactic forms in languages.

### Minimizing difficulty during production

Incremental production—the interleaving of plan and execution—works only if new plan segments can be developed at a rate that keeps up with execution. New plan development in turn relies on retrieval from long term memory, and when this retrieval fails or requires extra time, production is delayed or derailed. We next review three memory-related production biases that have substantial consequences for lexico-syntactic distributions in utterance form.

#### Easy first: a source of word order flexibility

As anyone who has been in a tip-of-the-tongue state knows, some words are more easily retrieved from memory than others. This fact has enormous influence on language form, because easily retrieved words and phrases tend to appear both earlier in utterances and at more prominent syntactic positions (e.g., sentence subject) than ones that are more difficult to retrieve (Bock, 1982; Tanaka et al., 2011). An Easy First bias in incremental production allows execution of utterances to begin early, starting with easily planned elements, leaving more time for planning of more difficult ones. “Easier” (also termed more accessible or available) words and phrases have been described as more frequent, shorter (both number of words in a phrase, and number of syllables in a word), less syntactically complex, more important or conceptually salient to the speaker, and previously mentioned (“given”) in the discourse (Levelt, 1982; Bock and Warren, 1985; Tanaka et al., 2011). There are enough different forces affecting ease of planning that the claims can seem circular: Easy entities are easy because they appear earlier in the utterance. However, the essential claim—that utterance planning difficulty affects speakers' choices of word order and sentence structure—gains external validity in several ways. First, difficulty stems from ease of retrieval from long term memory, and many of the factors that promote early positioning in an utterance plan also predict the early positioning and accuracy of word recall in verbal memory tasks, including word length, frequency, concreteness/imageability, givenness, and other factors (Bock, 1982). Second, other action and motor planning processes show these same Easy First tendencies. MacNeilage and Davis (2000) argued that the distributional regularities of consonant and vowels in infants' babbling and early words reflect infants' tendencies to order segments more easily articulated within the infant vocal tract before more difficult ones. Similarly, research in navigation shows an Easy First action ordering preference: when humans or animals have to visit several locations along a path, they typically begin with the nearest one (Gibson et al., 2007); if that nearest one is made difficult to reach because of obstacles, then more distant unobstructed locations tend to be visited first (Miyata and Fujita, 2011). Similarly, when humans are describing routes through a network, they also tend to begin by describing the simplest one first (Levelt, 1982). Third, Easy First biases in serial ordering inherently follow from computational models of action planning, in which alternative sub-plans compete for entrance into an action plan, via selection mechanisms in sequence planning (e.g., competitive queuing, Grossberg, 1978; Hartley and Houghton, 1996) or via gating functions of attention in models of cognitive control, in which more practiced/easier elements, which require less attention, precede more difficult ones in a developing plan (Botvinick and Cohen, submitted). Thus, the Easy First bias in language production is not a stipulative principle or language-specific phenomenon; instead it follows naturally from attested aspects of motor and action planning—that a plan precedes its execution, that planning is incremental, that the plan is hierarchical with subplans that must be ordered in some way, that plan development entails retrieval from long term memory, and that this retrieval varies in speed and accuracy.

These results suggest that the way that utterance planning unfolds over time has a substantial impact on the word orders and sentence structures that language users produce. Moreover, this work suggests a mechanistic basis for the observation that variation in language has functional importance (Givón, 1985): Word order variation, such as active/passive forms (*The noise startled the boy* vs. *The boy was startled by the noise*) and the English dative alternation (*give Mary a book* vs. *give a book to Mary*) allows producers the freedom to place easily retrieved elements early, permitting early execution of the plan, and allowing more time to plan the more demanding elements. Thus, in contrast to Jackendoff's (2002) suggestion that syntactic flexibilities are vestiges of ancient protolanguage, before syntactic constraints became more rigid, the PDC holds that word order flexibility has real value to language producers and emerges from action planning mechanisms that maximize fluency.

#### Plan reuse: a source of word order rigidity

Despite the enormous impact of Easy First on word order, it cannot be the whole story—people's utterances are not simply strings of words ordered by ease of retrieval from memory. Production also accommodates constraints on permissible word orders in a language. A second significant influence on utterance form also favors easy, more practiced plans, but in this case, what is easy is the abstract sentence plan itself rather than the word or phrase elements (sub-plans) within it. Producers have a conspicuous tendency to reuse recently executed utterance plans, so that the likelihood that a speaker utters a passive sentence, for example, increases if that speaker has recently heard, read, or uttered another passive sentence (Weiner and Labov, 1983; Ferreira and Bock, 2006). This tendency toward Plan Reuse (also called structural persistence or syntactic priming) persists over time and over other intervening utterances. The effect is argued to be not (or not only) the temporary activation of recent plans but rather a manifestation of long-term implicit learning of syntactic structure (cf. Branigan et al., 1999; Chang et al., 2006). On this view, language users are continually learning from their and others' language use; with every utterance, a syntactic plan becomes more likely to be used in the future. Thus, while the phenomenon is often described as one of short-term repetition, its learning basis links it to retrieval from long term memory—whereas Easy First refers to the effect of retrieval of individual words on word order, Plan Reuse effectively refers to the retrieval of the sentence structure itself. The two constraints jointly exert their influence on utterance form: Even in languages with very free word order, allegiance to favored structures (Plan Reuse) combines with Easy First in shaping utterance forms (Christianson and Ferreira, 2005).

The reuse of at least partially lexically-independent abstract plans is in some ways consistent with an autonomous syntactic representation independent of semantics (Chomsky, 1957), although the notion of adapting a prior syntactic plan to a new utterance, and the notion of sentences, phrases, and words as plans and sub-plans, are less consistent with the contrast in generative linguistics between a stored lexicon vs. generative syntax. Moreover, the reuse of abstract plans is not unique to language, as Plan Reuse appears in many non-syntactic and non-linguistic domains. Its effects are evident in recall from long term memory, in which people have a tendency to recall elements in the serial order in which they have frequently occurred in past experience (Miller and Selfridge, 1950). There is also increasing evidence for structured non-linguistic stimuli such as action sequences affecting subsequent production of certain sentence structures, suggesting that the re-use phenomena are not inherently linguistic (Allen et al., 2010; Kaiser, 2012). More broadly, similar Plan Reuse appears in many non-linguistic motor behaviors in humans and animals and is attributed to implicit motor learning. It is for these reasons that the reuse and adaptation of prior motor plans for subsequent action is thought to be a hallmark of motor planning and learning (Rosenbaum et al., 2007), and motor learning in the service of language appears to be no different. This point reappears in the section *Implications, Limitations, Future Directions*.

#### Reduce interference

Whereas Easy First and Plan Reuse stem from ease of recall from long term memory, Reduce Interference reflects properties of immediate memory instead of or in addition to long term recall. A classic finding in verbal and non-verbal short-term memory tasks is that the to-be-remembered elements interfere with one another in memory during the short interval between their presentation and recall, with increasing interference when the elements share similarity in sound, meaning, spatial location, or other dimensions (Conrad and Hull, 1964; Anderson, 1983). Because utterance plans are maintained before execution, it is not surprising that elements in the plan also interfere with one another. For example, when two semantically related nouns must be planned and uttered in close proximity (e.g., … *the couch and the chair…*), utterances take longer to plan and contain more errors than when this similarity is not present (Smith and Wheeldon, 2004). These effects may be a consequence of winner-take-all production: The path from conceptual message to word selection includes the partial (unconscious) activation of many alternatives (couch, sofa, loveseat, chair, furniture, etc.), and successful production requires that only one of these enter the utterance plan. Having then settled on *couch* and inhibited all others, the producer has additional difficulty—interference—when it becomes necessary to retrieve one of these inhibited options (e.g., *chair*; Tydgat et al., 2012). As with other examples discussed above, producers mitigate this interference via choices of utterance form (Gennari et al., 2012). A specific example is given in the next section, which considers how Reduce Interference interacts with Easy First and Plan Reuse.

#### The three factors in action

Easy First and Plan Reuse can pull in opposite directions, as Easy First promotes word order flexibility to allow easily-retrieved words before more difficult ones, whereas Plan Reuse promotes rigidity of word order via re-using previously uttered structures. Cross-linguistically, many distributional patterns of word orders reflect this tension, owing to different degrees of word order flexibility in different languages. In English and many other languages, passive structures such as (1b) are more common with animate subjects (*boy*) than with inanimate subjects like *window*. This pattern follows straightforwardly from the greater ease of memory retrieval of animate nouns than inanimate ones (Bock, 1982), and from the fact that the passive construction allows the easily-retrieved noun *boy* to be placed early in the sentence and in a prominent position (sentence subject).

1a. Active: The ball hit the boy hard, but he was OK.
1b. Passive: The boy was hit hard by the ball, but he was OK.

The three production planning factors make testable predictions about variation in passive use. First, if the relationship between noun animacy and active/passive form is the result of utterance planning pulled between Plan Reuse (favoring the more common Active form) and Easy First (favoring early mention of animates), then we would expect that animacy/Easy First effects on structure would be smaller in those languages (such as Slavic languages) that have a strong bias to use active forms. Results of this type are perhaps not surprising, because by definition, a strong allegiance to a single dominant word order to convey a particular message will allow less room for word order flexibility to accommodate ease of retrieval (Myachykov et al., 2011; Gennari et al., 2012)^2^. Second, utterance planning time should increase when these forces conflict compared to situations when they converge on the same form. This prediction is also supported (Ferreira, 1994). Third, if these structure and word order choices result from attempts to mitigate the computational demands of production planning rather than a specific discourse strategy to emphasize certain information for the comprehender, then we should also see effects of the third factor described above, Reduce Interference, interacting with the other two. This prediction is also supported; Gennari et al. (2012) found that when the agent and patient of an event are semantically similar (e.g. *boy, girl*), people more frequently describe the patient in passive structures such as *The girl was pushed (by the boy)*, in which the agent (*boy*) is demoted to a by-phrase or eliminated entirely in agentless passives (*The girl was pushed*). Here the system mitigates the demands of production by omitting, delaying, or demoting sentence elements that are affected by memory interference. These results suggest that while producers may sometimes (consciously or unconsciously) select a syntactic structure such as passives to convey a particular message, substantial variation in utterance form stems from the degree to which certain choices can reduce production difficulty for the producer. The results also suggest that both word order variation and word order rigidity have real value in production planning. Both the tendency to lead with easy elements and the tendency to adopt well-worn sentence types emerge from the nature of learning and retrieval from long term memory, in that highly frequent elements or well-practiced abstract plans, are preferred over more attention-demanding alternatives. On this view, implicit choices of both lexical items and sentence forms are shaped by the same memory-retrieval constraints.

Beyond increasing the fluency of an individual's utterances, these three production biases have another important consequence at the heart of the PDC that “individual-level behaviours result in population-level linguistic phenomena” (Scott-Phillips and Kirby, 2010, p. 1364). Summed over millions of utterances and many language producers, implicit production choices favoring less-difficult forms create dramatic statistical regularities in language usage linking conceptual messages, words, and sentence types. The next section relates this perspective to other approaches to language typology and universals and argues that a greater attention to production processes offers insight into questions about why some distributional patterns are more frequent than others.

## Distributional regularities and language typology

Functional linguists, language typologists, and historical linguists investigate the distributional regularities across the world's languages and their change over time, with one goal being the identification of significant cross-linguistic tendencies or linguistic universals that could illuminate the nature of human language^3^. Many functional linguists point to language use as a source of cross-linguistic patterns, meaning that languages tend to have (or develop over time) properties that serve the needs of language users (see Bybee, 2006, for review). The PDC's view is related, but it holds that specifically the needs of producers have the most direct effect on patterns of sentence structure. Indeed, this article is by no means the first to suggest that utterance planning processes in language production have an important role shaping language typology and historical change (e.g., Bock, 1982; Bock and Warren, 1985; Jäger and Rosenbach, 2008). Typologists have long observed that linguistic variation happens for a reason (e.g., Givón, 1985), and the production processes described above take steps toward a more mechanistic account of why word orders may vary from one situation to the next as a function the nature of retrieval from long term memory, the role of attention in gating competing processes in utterance planning, memory interference among entities in the utterance plan, and other factors. Moreover, the learning mechanisms of production that promote reuse of prior plans are an obvious candidate for informing accounts of language change (e.g., Bybee and McClelland, 2005). Despite this potential synergy between a more detailed study of language production mechanisms and language typology, there is relatively little consideration specifically of language production processes in the typology and universals literature (though see Bybee, 2006; also Jäger and Rosenbach, 2008, and associated commentaries). Why not? One obvious answer—lack of interaction between language typologists and language production researchers—is generally true but not fully satisfying, because it doesn't address why researchers in these areas feel little motivation to interact. Five more substantive assumptions underlying the disconnection are considered here, together with arguments for a rapprochement.

### Conceptual representations explain language form without language production mechanisms

Linguists have long noted that more conceptually salient elements (i.e., those more important to the producer and/or comprehender) occur early in utterances, such as the tendency for agents/animate entities to appear before undergoers of action/inanimate entities. These and related patterns have been attributed to varied forces, such as an Agent First principle in Universal Grammar (Jackendoff, 2002) and functional accounts in which elements that are salient in the discourse receive a prominent sentence position (Chafe, 1976; Goldberg, 2006). This latter position clearly shares a good deal with Easy First, but the production account goes beyond an appeal to salience in important ways. First, the Easy First bias in production grounds the conceptual salience effect in ease of recall from long term memory. Since salience itself is not acting directly on prominence but rather via ease of recall, this approach correctly predicts that other non-salience factors affecting ease of recall (e.g., word length) can also affect word order. Second, the incremental nature of motor planning for production explains why the privileged location for easily recalled entities is early in the utterance plan rather than saving the easiest for last. And third, filtering conceptual salience through the production system accounts for situations in which communicative goals influence salience (via task-specific allocation of attention) rather than the other way around (Kuchinsky et al., 2011). Thus, “salience” does affect word order, but it gains external validity via an understanding of language production, memory recall, action planning, and motor control.

### Production gets typology wrong

In English and many other languages, shorter words and phrases tend to precede longer ones, which has been attributed to Easy First (Stallings et al., 1998). However, Hawkins (2004) describes some notable exceptions in Japanese and other “head-final” languages, casting doubt on production as a source of typological patterns of word order: “The preference for long before short in Japanese is not predicted by current models of language production, all of which are heavily influenced by English-type [languages]” (p. 110). Hawkins is correct that language production researchers have investigated relatively few languages, and this concern is compounded by psycholinguists' tendency to pursue narrow, controlled studies focusing individually on Easy First, Plan Reuse, or Reduce Interference, with relatively little attention to the fact that multiple factors can contribute to retrieval from memory and thus word order. Things are improving on both these fronts, and more recent production accounts do investigate the origin of opposing effects of phrase length in English and Japanese (Yamashita and Chang, 2001), including a computational model of language production that develops Short-before-Long preferences when trained with English input and learns a Long-before-Short preferences when exposed to Japanese input (Chang, 2009). Analyses of the model's performance in the two language environments point to Plan Reuse as one important force in developing the ordering preferences, in that tendencies for ordering object and recipient noun phrases reflect the adaptation of plans from more common sentences with only one noun phrase. Thus, production work may have been late to the party here, but if Chang is correct about the role of learning mechanisms and Plan Reuse, then his mechanistic account of utterance planning will prove central to these cross-linguistic differences.

### The grammar-performance disconnect

The value of distinguishing linguistic competence (knowledge: the grammar) and performance (use) has long been a source of debate within linguistics (Newmeyer, 1998; Jackendoff, 2007) and is beyond the scope of this article. Two trends are worth noting, however: First, some linguistic approaches increasingly view grammar itself as a graded representation emergent from experience with language tokens (Bybee, 2006; Bresnan et al., 2007), and this position (whether or not “grammar” is invoked in the explanation), is central to recent production-based accounts of word order variation (Kuperman and Bresnan, 2012). Second, whatever one's position on the nature of grammar, utterance planning itself clearly shapes utterance form, and as such, production merits more attention in typology if only to better attribute distributions to the work of grammar vs. performance.

Relatedly, the culture of controlled laboratory studies in language production is at odds with typologists' interests in the broad sweep of cross-linguistic patterns. These trends are changing in several ways. First, researchers are increasingly investigating the link between individual-level phenomena (as studied in many language production studies) and the population-level phenomena, where interactions among many individuals affect language form over time (e.g., Scott-Phillips and Kirby, 2010). Clearly the PDC will benefit from improved understanding of individual-population interactions. Second, there is increasing use of large corpora as a form of production data being brought to bear on typological issues (e.g., Piantadosi et al., 2012), and a move by some psycholinguists to adopt information theoretic approaches to language performance, in which accounts of language distributions invoke notions of communicative efficiency (e.g., Jaeger and Tily, 2011). This perspective obviously has clear overlap with functional linguistic accounts of typology, but from the PDC perspective, it's important to incorporate a detailed account of the cognitive control and memory demands of production planning into accounts of “efficiency” and why some distributional patterns are more common than others. It remains to be seen how this tension between information theoretic and more mechanistic accounts plays out.

### Typology and universals reflect acquisition, not production

Whereas many functional linguists consider adult language use as key to understanding language universals and change (Bybee, 2006), others point to the child learner as the engine of change, either via the application of Universal Grammar (e.g., Lightfoot, 1999) or via innate learning biases in the child (Hudson Kam and Newport, 2009; Culbertson et al., 2012). From the PDC perspective, this distinction between acquisition and use is a false one, because learning in production (and comprehension) never stops; there is not first a phase of acquisition and later one of use (Seidenberg, 1997; Chang et al., 2006)^4^. Learning specifically in the service of production may be particularly important in understanding the nature of language typology and change, first because utterance planning requires memory retrieval, which is itself a powerful determinant of further learning (Karpicke, 2012). Thus, each production event should have on average a stronger effect on subsequent learning than each comprehension event, owing to the greater retrieval demands in production. Second, production is winner-take-all, meaning that someone who has perceived variable input in the past must commit to only one form for any given utterance, with potential consequences both for subsequent distributional patterns and for learning over one's own productions^5^. Thus, more attention to learning for production could inform current unknowns concerning hypothesized links between acquisition and typology: “*why* learners acquire certain types of patterns more easily than others (and why languages therefore more commonly exhibit these patterns)” (Aslin and Newport, 2012, p. 174).

A central assumption in the role of learning biases in language acquisition and typology is that child learners are biased to make input more regular, as when a child uses *holded* for the past tense of *hold* rather than *held*. Considered from the point of production, the use of *holded* vs. *held* is an implicit choice of utterance form over available options, similar to saying *cat* vs. *kitty* or a passive vs. active sentence. However, a child who utters *holded* may have never encountered it before; what could cause a bias toward producing a form that's generally unattested in the input? Ease of production is a good bet, both because it is such a powerful force in adult language production and also because child utterances are full of omissions and other simplifications that reduce utterance difficulty despite being unattested in the adult input. The production force that promotes overregularization is Plan Reuse, where the abstract plan here is the regular inflection, which becomes increasingly common as the child learns more verbs (Rumelhart and McClelland, 1986). On this view, children's novel forms such as omitting an initial unstressed syllable (e.g., saying *nana* for *banana*) and *holded* for *held* are not distinct phenomena but are instead both examples of the strong influence of Plan Reuse, reflecting the dominance of first syllable stress in English nouns (Echols and Newport, 1992) and the dominance of the regular inflectional paradigm in English.

This example argues for a different approach to considering the role of learning in language typology and change, namely shifting the question from “Why are some forms more easily learned by the child?” to a child version of the same question we've asked about adults: “Why are some forms more often produced?” Viewing the overregularization effect as owing to production choices is broadly consistent with accounts in which the effects of experience with individual words and with the regular paradigm (the plan) vary with the amount of prior exposure (Rumelhart and McClelland, 1986). This approach also yields the correct prediction that children who overregularize may nonetheless comprehend the irregular form that they don't produce (Clahsen et al., 2007; Ramscar and Yarlett, 2007). These results suggest there may be real value in considering the child's utterance planning demands in phenomena that have previously been attributed to more general learning biases or Universal Grammar. This position is clearly not anti-learning but rather an argument for considering what the learning is for.

### Language is tailored to the comprehender

In arguing for a central role for production in shaping language form, the PDC does not deny that other forces may also influence form. However, if these other forces turn out to be extremely powerful, they could erode the PDC's claim for the centrality of production in shaping language form. One alternative force shaping utterance form is *audience design*, the idea that language producers tailor their utterances to accommodate the needs of the comprehender. Clearly producers do make adaptations to the needs of the perceiver; the act of language production is itself an accommodation, in that the producer is adapting to the fact that the perceiver is not a mind reader and needs an overt linguistic signal. However, this adaptation is inherently limited, because the producer is also not a mind reader and therefore cannot fully know what needs the perceiver has. Thus, both audience design and production-driven utterance choices likely exist in parallel.

Attributing aspects of utterance form to producer needs (as in the PDC) vs. audience design is a complex undertaking, for several reasons. First, the most obvious potential evidence for audience design—that comprehenders benefit when producers use certain forms and find other forms difficult—turns out not to be that useful. Perceivers routinely benefit from statistical patterns in their input that have no audience design; we can predict the trajectory of a bouncing ball, but the ball does not aim to help our tracking. Moreover, producer variation that might seem designed for an audience can instead have purely production-centered explanations. Consider the phenomenon of phonological reduction, in which speakers introduce a word into a discourse with a fairly careful articulation and later re-mention it in a less precise “reduced” form (Fidelholtz, 1975). Listeners clearly have learned these reduction patterns and benefit from them (Dahan et al., 2002), but this benefit does not mean that phonological reduction is designed to help the listener. Indeed, reductions of this sort are an inherent consequence of motor learning and practice generally, including in motor behaviors with no audience (Müller and Sternad, 2004).

A second complicating factor is that audience design is not cost-free: accommodating a comprehender will itself impose demands on the producer. The producer must work to identify perceiver needs, and as this task becomes more difficult (e.g., requiring more elaborate inferencing), the amount of audience design in the utterance declines (Horton and Keysar, 1996; Bard et al., 2007). Thus, we can't talk about whether utterance form comes from perceiver *or* producer needs, because perceiver accommodation inherently creates demands on the producer.

Third is the existence of accommodation in the other direction, in that perceivers accommodate the needs of the speaker (Duran et al., 2011). Some obvious examples, many of which are not always identified as producer accommodation, include various forms of phonetic adaptations (Kraljic et al., 2008) and ambiguity resolution at lexical, syntactic, and other levels (MacDonald et al., 1994). Perceivers may often be good at accommodation because they have direct information about the producer's perspective from the utterance itself. Indeed, recent information-theoretic analyses have suggested that overall communicative efficiency is not optimal when the producer is maximally clear and redundant, which would make utterances longer and more carefully articulated than the perceiver needs. Instead, communicative efficiency is higher when the producer uses short ambiguous words and permits phonological reduction and substantial additional ambiguity (Piantadosi et al., 2012). This arrangement works because comprehenders are so good at ambiguity resolution and other forms of speaker accommodation. Such results turn the notion of audience design on its head: Tuning the conversational interaction primarily to the producer's needs, and letting the perceiver accommodate the producer, is in a broad sense *a form of audience design*: The producer adopts utterance forms mitigating difficulty and maximizing fluency so that the conversation proceeds efficiently, without bogging down the process with more redundancy than the perceiver needs.

These considerations suggest that audience design is not incompatible with producers' implicit choice of utterance forms that mitigate production difficulty. On this view, the key question is not whether there is audience design (there is), but rather how distributional patterns emerge from the specific computational demands of language production as executed by producers who have a communicative goal. Because audience design contributes to the computational demands of utterance planning, researchers who study the mechanisms of production planning should accommodate the audience design literature more fully, and vice versa.

### Summary

This highly selective discussion contains almost no typological data and omits many issues in the current literature. The aim is not to review PDC contributions to typology (that section would be very short indeed) but rather to suggest that there is sufficient promise for cross-disciplinary interaction, specifically that the computational demands of utterance planning, and producers' attempts to minimize them, should be investigated further as an important driving force in cross-linguistic language typology and change. Though not elaborated here, the reverse is also true: Work on the statistical distributions in the world's languages, and the way that languages change over time, can inform psycholinguistic accounts of language processes (e.g., Feist, 2010; Culbertson et al., 2012).

## Comprehension consequences in the PDC

Having reviewed implicit choices of utterance forms and consequences for distributions in the language input, we now consider what comprehenders do with these distributions. The next section addresses what language users learn about distributional regularities, and the two sections after that review two classic examples of sentence comprehension phenomena, for which popular theories have attributed comprehension behavior to architectural properties of the comprehension system—in effect, that comprehension works the way it does because innate parsing biases make it so. By contrast, the PDC approach suggests that the comprehension results reflect distributional regularities in the language, which themselves can be traced to the joint actions of Easy First, Plan Reuse, and Reduce Interference shaping the forms of utterances during utterance planning.

### Distributional regularities and prediction in comprehension

Linguistic signals unfold over time, creating long distance dependencies, where the interpretation of some input is dependent on previous or upcoming parts of the signal. Integrating over these dependencies involves use of probabilistic information in both forward and backward directions to settle on the most likely interpretation of the input. In the backwards direction, recently-encountered information allows further refinement of the earlier input (MacDonald, 1994), with some effects strong enough to affect what perceivers report they hear (Warren and Sherman, 1974; Connine and Clifton, 1987; Mack et al., 2012). Use of statistical dependencies in the forward direction does not necessarily entail exactly predicting upcoming words but rather generating general expectations about grammatical category, gender, and other properties that greatly narrow the scope of possibilities (Van Petten and Luka, 2012). The notion that comprehenders are generating expectations for upcoming input has been a controversial one, as it has not always been clear that predictions could be sufficiently constraining or efficiently computed (MacDonald and Seidenberg, 2006). However, partial predictions can emerge naturally from a system operating under time pressure (Allen and Seidenberg, 1999), and predictions may arise from many correlated language statistics, so that low levels of the linguistic signal, such as acoustic or orthographic form, can provide extremely early probabilistic information about grammatical category or syntax even in advance of word recognition (Dikker et al., 2010). Moreover, parts of the signal that have predictive value for upcoming percepts not only speed the processing of the predicted elements downstream but may themselves be processed more rapidly than uninformative signal, owing to cortical feedback mechanisms gating attention toward potentially informative input (O'Brien and Raymond, 2012). Together, this work reflects a point that's evident in information theoretic accounts of language processing but hasn't consistently penetrated other comprehension approaches, that there is *always* ambiguity in the language signal as it unfolds over time, and uncertainties about both the upcoming and the recently encountered signal are a source of processing difficulty (e.g., Hale, 2006; Levy, 2008). Use of distributional regularities to reduce this uncertainty is a powerful advantage in comprehension, and the next two examples suggest how a deeper appreciation of this fact, together with an understanding of how production processes create certain distributional regularities, reframes our understanding of sentence comprehension.

### Reinterpreting parsing principles: verb modification ambiguities

A pernicious type of sentence ambiguity, the verb modification ambiguity, is shown in (2), in which an adverbial phrase could modify one of two different actions described in the sentence. Example (2a) shows a fully ambiguous structure, (2b) shows an example in which verb tense disambiguates the sentence in favor of a the *local modification* interpretation in which the adverb *yesterday* modifies the nearest verb *left* rather than the more distant phrase *will say*, and (2c) is an example of distant modification, in which *tomorrow* modifies the distant verb, *will say*.

2a. Verb Modification Ambiguity: John said that his cousins left yesterday.
2b. Local Modification: John will say that his cousins left yesterday.
2c. Distant Modification: John will say that his cousins left tomorrow.

English comprehenders greatly favor local modification (2b) over distant modification (2c). This pattern is often thought to arise directly from innate parsing or memory biases to favor local phrasal relationships over long distance ones, variously formulated as Right Association (Kimball, 1973), Late Closure (Frazier, 1987), and Recency (Gibson et al., 1996). A key assumption has been that these parsing principles operate on purely syntactic representations without lexical content (e.g., Frazier, 1987). This approach accorded well with the fact that, with few exceptions (Fodor and Inoue, 1994; Altmann et al., 1998), the lexical content of sentences like (2) has minimal effect on English speakers' strong bias in favor of local modification, making verb modification ambiguities the best available evidence for lexically-independent innate parsing algorithms.

As Table 1 summarizes, the PDC approach accounts for the local interpretation biases without innate parsing algorithms. Instead the effects stem from learning over the distributional regularities in the language, which in turn stem from the biases of producers to favor certain sentence forms that minimize production difficulty.

**Table 1:** **Production-Distribution-Comprehension (PDC) account of greater comprehension difficulty for ambiguities resolved with distant modification (2c) than with local modification (2b)**.

**PDC STEPS**	
1.	**Production:** Easy First, where shorter phrases precede longer ones, discourages production of ambiguous structures like (2a) with intended distant modification, and instead promotes production of other forms to convey the same message (MacDonald, 1999; MacDonald and Thornton, 2009).
2.	**Distribution:** As a result, ambiguous sentences with intended distant modification are much rarer than ambiguous sentences resolved with local modification (Sturt et al., 2003; MacDonald and Thornton, 2009).
3.	**Comprehension:** The comprehension patterns reflect the language statistics in Step 2: Overall, the rarer distant modifications are harder than the more common local modification sentences (Altmann et al., 1998; MacDonald and Thornton, 2009). However, a subtype of verb modification ambiguities don't violate Easy First in their distant modification form, owing to the relative length of phrases in these sentences. These are readily produced by speakers who intend distant modification, are common in the language, and are easily comprehended (MacDonald and Thornton, 2009).

In Step 1 in the table, the Easy First production bias discourages production of distant modification sentences like (2c) because more easily planned alternatives exist. In (2c), a relatively long phrase (*that his cousins left*) precedes a short one (*yesterday*), but Easy First promotes a short-before-long phrase order, as in *John said yesterday that his cousins left*, or *Yesterday, John said that his cousins left*. Step 2 identifies the distributional consequences of speakers avoiding utterances like (2c): Ambiguous sentences like (2a) typically have a local modification interpretation like (2b). Comprehenders are extremely sensitive to these statistics (Step 3), and they have difficulty comprehending largely unattested forms like (2c), but they readily comprehend a special type of distant modification sentences that don't violate Easy First and that do exist in the language. These results suggest that rather than an innate comprehension bias for local modification, perceivers have a learned bias toward what has happened in the past, and that this prior linguistic experience owes to aspects of production planning.

This claim for the role of past experience on subsequent comprehension processes is at the heart of constraint-based accounts of language comprehension, which have been applied to many other syntactic ambiguities (see MacDonald and Seidenberg, 2006, for review). The added value of the PDC is (a) a greater emphasis on the role of learning probabilistic constraints, and (b) an account of the production basis of the language distributions that people learn and use to guide comprehension. Extending the PDC to other syntactic ambiguities is ongoing; the approach holds promise because (a) these ambiguities turn on the relative frequency of alternative uses of language, which can be readily learned from input (Wells et al., 2009), and (b) certain production choices are known to affect syntactic ambiguity. For example, variation in availability of genitive forms (*the professor's daughter* vs. *the daughter of the professor*) in English vs. other European languages affects the distribution of noun modification ambiguities and their interpretation in these languages (see Mitchell and Brysbaert, 1998, for review and Thornton et al., 1999 for constraint-based studies of cross-linguistic similarities and differences). Similarly, producers manage production demands through the use of optional words (e.g., Ferreira and Dell, 2000), which have substantial effects on ambiguity, the distribution of form-meaning pairings, and consequent experience-driven ambiguity resolution processes. Thus, the PDC prediction is that *all* syntactic ambiguities can ultimately be traced back to producers' implicit utterance choices (many in the service of reducing utterance planning difficulty), the consequent distributions in the language, and comprehenders' learning over those distributions.

### Reinterpreting syntactic parsing and working memory burdens

The next example, relative clause interpretation, repeats the PDC argument—mitigating production difficulty leads to utterance choices that lead to distributional regularities that lead to comprehension patterns. Relative clauses nonetheless merit detailed attention, first because they illustrate complex interactions of all three production biases, and second because they have played an outsized role in theories of both syntax and language comprehension, so that a revision of traditional claims has substantial consequences.

#### The relative clause trifecta: recursion, competence-performance, and working memory

Relative clauses are noun modifiers that include a verb, as in examples (3a,b). In (3a) *the ball* is being modified by the bracketed relative clause; because *the ball* is the object of the relative clause verb (*threw*), this structure is called an object relative (or center-embedded) clause. A subject relative clause is illustrated in (3b), where *woman* is the subject of the relative clause verb *yelled*. These two examples seem pretty innocuous, but in fact subject and object relative clauses have played a central role in defining the differences between language competence and performance in generative linguistics, and they also have had an enormous impact in essentially every area of comprehension research, from acquisition, to adult comprehension, to studies of aphasia and other language impairments.

3a. Object relative: The ball [that I threw to Harold] went over his head and broke a window.
3b. Subject relative: The woman [who yelled at me] said I'd have to pay for the broken glass.

The origin of relative clauses' importance can be traced to claims by Miller and Chomsky (1963) concerning reasons behind the comparative difficulty of subject relatives vs. center embedded object relatives. Chomsky and Miller (1963) observed that the repeated recursive operation of embedding one object relative inside another one yields an uninterpretable sentence; their example was *The rat [the cat [the dog chased] killed] ate the malt*. Miller and Chomsky (1963) viewed the difficulty of these sentences as following from a distinction between linguistic competence and ability to use that knowledge—linguistic performance. They argued that while linguistic competence (here, recursion) is infinite, performance, specifically the ability to use this knowledge to comprehend center embedded structures, is constrained by limitations on short-term memory capacity (Miller, 1956). In the case of object relative clauses, the memory burden stems from the multiple incomplete noun-verb dependencies arising as the sentence unfolds, so that the comprehender must first anticipate a verb for each noun (*the rat the cat the dog*) and hold these unintegrated nouns in memory, and then when the verbs are encountered (*chased killed ate*), associate them appropriately with the nouns (Wanner and Maratsos, 1978; Gibson, 1998). By contrast, the more comprehensible English subject relatives interleave nouns and verbs, reducing the memory burdens: *The dog [that chased the cat [that killed the rat]] howled*.

It is difficult to overstate both the impact of Miller and Chomsky's analysis and the subsequent reach of relative clauses into virtually all corners of language comprehension research. Several additional factors have contributed to the central position of relative clauses in theories of memory and language use. First, relative clauses are widely held to be syntactically unambiguous (Babyonyshev and Gibson, 1999), so that comprehension difficulty can't be attributed to ambiguity resolution processes. Second, subject and object relatives can be made to differ by only the order of two phrases, as in the order of *the senator* and *attacked* in (4a,b), so that researchers can contrast comprehension of sentences for which the lexical content seems perfectly matched. The vast majority of a very large number of studies in English and many other languages, across children, adults, individuals with brain injury, disease, or developmental atypicality, show that object relatives are more difficult than their matched subject relatives (see O'Grady, 2011, for review). The logic here seems perfectly clear: Because the difference in difficulty can't be ascribed to lexical factors or ambiguity resolution, it must reflect *purely syntactic* operations and the memory capacity required to complete them (Grodner and Gibson, 2005).

4a. Object relative: The reporter [that the senator attacked] admitted the error.
4b. Subject relative: The reporter [that attacked the senator] admitted the error.

This competence-performance account of working memory overflow in relative clause comprehension continues as the dominant perspective in linguistics, language acquisition, adult psycholinguistics, and communicative disorders, despite criticisms of each of the components of this argument. These criticisms include evidence that multiply center-embedded sentences need not be incomprehensible (Hudson, 1996), comprehension difficulty is strongly influenced by the words in the sentence and therefore cannot reflect purely syntactic processes (Traxler et al., 2002; Reali and Christiansen, 2007), object relatives do contain a non-trivial amount of ambiguity directly related to comprehension difficulty, again refuting the assumption that relative clauses provide a pure measure of syntactic difficulty (Gennari and MacDonald, 2008), the degree of prior experience with object relatives predicts comprehension success in children and adults, a result not captured by memory overload approaches (Roth, 1984; Wells et al., 2009), people's comprehension capacity for recursive structures is more accurately described by a system in which working memory is inseparable from linguistic knowledge than by one with separate competence and performance (Christiansen and Chater, 2001), and that cross-linguistically, relative clause complexity does not always predict comprehension difficulty (Lin, 2008; Carreiras et al., 2010). The resilience of memory overflow accounts in the face of these myriad challenges in part reflects the essential usefulness of the constructs of working memory capacity and competence-performance distinctions in cognitive science. However, a second factor is that there has been no really compelling alternative account that captures both the subject-object relative asymmetry as well as these other phenomena. The PDC approach aims to provide exactly this.

#### The relative clauses that people produce

Insight into why object relatives are hard requires noting producers' available choices, specifically that there are two ways to describe the patient/theme of some action with a relative clause, either an object relative (5a) or a passive relative (5b; curly brackets indicate the optional passive “by-phrase” identifying the agent of the action).

5a. Object relative: The boy/toy [that the girl splashed] was dripping wet.
5b. Passive relative: The boy/toy [that was splashed {by the girl}] was dripping wet.

**Figure 1:**
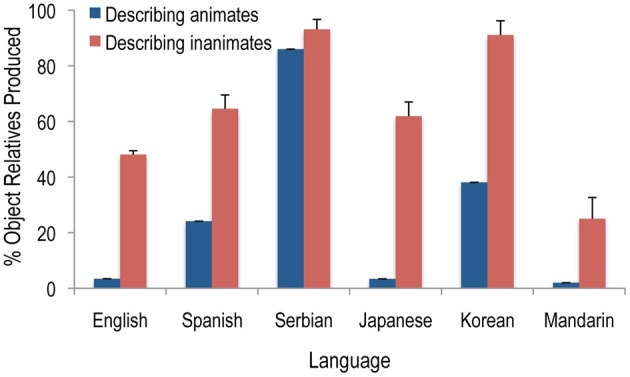
**The frequency with which object relative clauses are produced to describe animate and inanimate entities in a picture description task, calculated as a percentage of all relative clauses produced.** The English, Spanish, and Serbian data are from Experiments 1a, 2, and 3 of Gennari et al. (2012), respectively. The Japanese data are from Montag and MacDonald (2009), Korean from Montag et al. (in preparation), and Mandarin from Hsiao and MacDonald (in preparation).

Step 1 in Table 2 describes how producers' use of object relatives vs. passive relatives is shaped by the joint action of Easy First, Plan Reuse, and Reduce Interference. When English producers are describing something inanimate (e.g., *toy*), they readily produce object relatives like (5a), but they almost never do this to describe something animate (*boy*). Instead, they utter passive relatives like (5b). This pattern is not limited to English; my colleagues and I have investigated relative clause production in six languages, which differ widely in word order in main and relative clauses, the amount of case marking on nouns, the availability of alternative structures to express this same message, and many other properties. Figure 1 shows that in all six languages, object relatives are produced less often when describing animate entities than inanimate ones^6^.

**Table 2:** **PDC account of greater comprehension difficulty for object than subject relative clauses (citations refer to English results)**.

1.	Object relatives (5a) are common when the noun being described is inanimate (*toy*) but are avoided when the relative clause describes something animate (*boy*), passive relatives (5b) are produced instead (Montag and MacDonald, 2009; Gennari et al., 2012). These patterns owe to at least three production biases: **Easy First**: animate nouns are conceptually prominent and easily retrieved from memory, leading to their position in early or prominent sentence positions. The passive relative (5b) allows the described noun to be in the prominent subject position of the relative clause. **Plan Reuse**: the rate of passive relatives varies with the viability of passives in the language more generally, reflecting the reuse of passive forms from other sentence types (Montag and MacDonald, 2009). **Reduce Interference**: there is more interference between conceptually similar entities [*e.g. boy/girl* in (5)] than when an animate entity (*girl*) acts on an inanimate one (*toy*). This interference can be reduced by omitting the agent in the utterance plan, which is possible in passive forms (5b), but not in object relatives (5a). The higher the conceptual similarity between sentence participants in the event to be described, the more speakers produce passive agent-omission relative clauses (Gennari et al., 2012).
2.	People readily learn these correlations between animacy and relative clause type (Wells et al., 2009).
3.	Comprehenders who encounter the start of a relative clause have very different expectations for how it will end, depending on whether something animate or inanimate is being described, with consequences for comprehension: When relative clauses describe something inanimate like *toy*, English speakers rapidly anticipate an object relative (5a); for animates (*boy*), object relatives are vanishingly rare and are not expected by comprehenders (Gennari and MacDonald, 2008). The less producers are willing to say an object relative to convey a particular message, the less comprehenders expect one, and the more difficult the comprehension is when a sentence in fact turns out to contain an object relative clause (Gennari and MacDonald, 2009).

Figure 1 also shows large cross-linguistic differences in the overall rate of object relative use. The reasons behind these differences are quite complex and of course reflect important topics in language typology. Some variation in overall tolerance for object relatives appear to reflect Plan Reuse and the viability of passives in main clauses in a language (Montag and MacDonald, 2009), and other important factors may include whether the language has other utterance forms that speakers might use beyond object and passive relatives, and the extent to which nouns are marked for case and the flexibility of word order in a language, both of which appear to modulate the degree interference between the agent of the relative clause and the entity being described by an object relative clause (Gennari et al., 2012). There are undoubtedly other complex influences as well.

Although we are just beginning to understand the factors behind the patterns in Figure 1, it is clear that speakers' very different choices for animate-describing and inanimate-describing relative clauses have robust effects on the distributional regularities in these languages. Steps 2–4 of Table 2 show the cascade of consequences of these choices—comprehenders rapidly learn the robust form-meaning correlations (Step 2), and they bring this information to bear in comprehension, such that they expect object relatives where they're commonly produced but are surprised by them in unexpected environments, leading to comprehension difficulty (Step 3). The vast majority of studies demonstrating object relative processing difficulty have used materials in which something animate is being described—the very situation that producers avoid and that comprehenders have learned not to expect. Together, the steps in Table 2 suggest that object relative clause comprehension is simply another example of ambiguity resolution—comprehenders are “led down the garden path,” as the saying goes in parsing research, by relying on past experience that leads to incorrect expectations for these unusual sentences, and the results do not reflect any pure effect of syntactic complexity on comprehension (Gennari and MacDonald, 2008)^7^.

On this view, relative clauses, which have been central to current conceptions of memory and language use in virtually every subfield of psycholinguistics, turn out to be wholly unsuited for that role, as they are not unambiguous, and their comprehension reflects detailed knowledge of correlations between words and structures, not abstract syntactic representations. What then becomes of working memory limitations as a source of comprehension difficulty, particularly within Miller and Chomsky's (1963) competence-performance claims for infinite recursion limited by working memory? The short answer is that researchers may further debate competence-performance distinctions, but relative clauses should no longer be offered as evidence of overflow of syntactic memory representations that limit infinite recursive capacity. A more precise answer about implications of the relative clause work requires closer attention to what working memory is and isn't. In saying that the PDC account refutes claims for working memory limitations in sentence comprehension, my colleagues and I do not mean that working memory doesn't exist—to the contrary, a prime reason why language users track the statistics of the language and use them to anticipate upcoming input is precisely because language comprehension requires significant memory capacity, and generating expectations for likely outcomes reduces these burdens. However, we do reject the notion that people's working memory capacity can be described as a performance limitation *independent* of their linguistic knowledge/competence (MacDonald and Christiansen, 2002; Acheson and MacDonald, 2009; Wells et al., 2009). Our position reflects broader trends linking working memory and long-term knowledge (Cowan, 2005), emergent from the temporary maintenance needs of other cognitive processes (Postle, 2006). Specifically for relative clauses, comprehension capacity varies with long term knowledge of these structures, derived from experience. Language producers provide some kinds of experiences (some kinds of relative clauses) more than others, with consequences for language distributions, learning over those distributions, and for the memory demands needed to comprehend these structures—the memory capacity and experience cannot be separated. Of course computational limitations, including memory limitations, are also at the heart of the PDC argument for why producers prefer some utterance forms over others, but this does not mean that the competence-performance distinction can simply be shifted to production, because again, linguistic working memory, specifically the capacity to produce certain utterance forms, is not separate from long term linguistic knowledge or experience (Acheson and MacDonald, 2009).

### Summary

The two cases reviewed here, verb modification ambiguities and relative clauses, exemplify the PDC's point that an understanding of production choices in a language is critical for understanding comprehension. That idea has been implicit in non-syntactic comprehension work for decades (e.g., in lexical frequency effects on word recognition, in that frequency is inherently an effect of experience and ultimately producers' word choices), but it's quite another thing to claim that we could abandon many of the special-purpose syntactic interpretation mechanisms common in the parsing literature if we understood sentence production better. It will take some time to test this view in other constructions and languages, but in the meantime, the availability of extensive language corpora in many languages permits comprehension researchers to examine the relationship between production patterns (in the corpus) and comprehension behavior, even if they have not yet investigated the production pressures that create the distributional regularities that are observed in a corpus. The PDC suggests that it is essential to investigate such linkages before declaring that comprehension behavior owes to highly specific design features in the language comprehension system.

## Implications, limitations, future directions

The PDC begins with something utterly uncontroversial, that language production is hard. The next step is no less obvious to production researchers, that language producers try to make things easier, and that their attempts affect the form of the utterances they produce. From there we get into somewhat more controversial territory that (a) producers' choices of utterance forms, repeated through the population, have a significant role in explaining language typology and change over time, and (b) language users learn these statistical patterns and rapidly use them to interpret new input. There are aspects of all of these ideas in the literature, but the PDC is greater than the sum of these parts in suggesting that the downstream influences of production processes are so strong and so pervasive that we must take production processes into account in developing theories of language form, change, and comprehension.

One of the ways that the PDC is different from related ideas is its emphasis on a specifically *mechanistic* account of language production. It is certainly not wrong to appeal to more abstract notions of communicative efficiency in accounting for producers' choices of utterance forms (e.g., Jaeger and Tily, 2011; Piantadosi et al., 2012), but the PDC can offer something more to the extent that it draws on the mechanisms of memory retrieval, attention, serial order maintenance, and motor planning in understanding what is more vs. less efficient. Similarly, Bybee (2006) and others make important claims that language use, broadly construed, underlies language typology and change, but the PDC aims to be more specific: Language producibility, more than learnability or comprehensibility, drives language form. The reasons for this claim again invoke mechanistic accounts of language production to explain what is difficult, how producers manage that difficulty, and how they are the primary controllers of utterance form. There's a great deal of work remaining in order to realize this goal of a mechanistic account of language production, including extensions beyond the lexico-syntactic focus of this article. Working toward a more mechanistic account is important because links to memory, action planning, and other non-linguistic domains can ground the PDC approach in broader cognitive processes and avoid potential circularities among what is efficient, common, easy, salient, and other constructs that are invoked in many accounts of language form and use.

This linkage between action planning and the mechanisms of language production has several intriguing implications for the way language researchers view language form and use. First, an implication for psycholinguistics: For decades, experience-based sentence comprehension research has emphasized the non-independence of lexical and syntactic representations during the comprehension process (e.g., MacDonald et al., 1994). By contrast, language production and motor/action planning more generally rely on abstract high-level plans that appear quite independent from the elements in the plan. Understanding how the demands of comprehension and production integrate lexical and more abstract hierarchical representations is an important challenge as these fields move forward. One possibility is that comprehension processes may draw on covert language production processes and other aspects of non-linguistic motor planning (Pickering and Garrod, 2007). If so, production may be doubly intertwined with comprehension, both in the PDC's view of production mechanisms generating the statistics of language forms that drive comprehension, and also Pickering and Garrod's argument for covert production processes in the service of comprehension.

Second, the link between language and action planning has implications for how we view language itself. An enormous literature considers how language is distinct from non-linguistic cognition (see Newmeyer, 1998; Jackendoff, 2002, 2007, among numerous others), but the PDC may be able to contribute to the discussion. There has been little work to date investigating the commonalities and differences between the abstract hierarchical plans that underlie sentence production and those that underlie non-linguistic motor behavior. To the extent that such commonalities exist, they could suggest that syntax, at least as it is realized in creating utterances, has a potential homologue in non-linguistic systems and therefore is not something that distinguishes language from other cognition. However, linguistic utterances clearly differ from other actions in that they have both a goal (e.g., to communicate) and a meaning, while complex actions have a goal (e.g., to make coffee), and a hierarchical plan to realize the goal, but no inherent meaning. This meaning and its interplay with utterance form, meted out over time as the language is planned, produced, and comprehended, would seem to be a critical aspect of what makes language unlike non-linguistic cognition. Again, work toward a mechanistic account of how language is planned and uttered may have consequences well beyond the field of language production.

### Conflict of interest statement

The authors declare that the research was conducted in the absence of any commercial or financial relationships that could be construed as a potential conflict of interest.

## Footnotes

^1^“Utterance” here refers to all modalities (speaking, writing, signing). Each modality's unique production demands should influence the distribution of forms in that modality, but those effects aren't discussed here.

^2^See Bresnan and Ford (2010), Stallings et al. (1998), and Wasow (1997), for other examples of the tension between Easy First and Plan Reuse, though not using these terms. The reasons why one language would have freer word order than another is of course something to be explained within any perspective. In production based approaches, large-scale corpus studies should prove useful, as in investigations of the rigidity of use of dative constructions in American vs. Australian English (Bresnan et al., 2007; Bresnan and Ford, 2010).

^3^This statement dramatically simplifies functional, historical, and typological linguistics as well as the debate about whether there are truly universals of language or merely asymmetries in the distribution of language features in the world's languages (Evans and Levinson, 2009).

^4^An approach favoring continuity between acquisition and use raises questions about discontinuities, specifically sensitive period effects in language acquisition. Proactive interference approaches, in which prior learning affects the rate of subsequent learning (Seidenberg and Zevin, 2006), suggest that child vs. adult language users may have different effects on language form, but not because of a distinction between acquisition and use.

^5^Frequency boosting, in which a slightly dominant form strengthens over time (Singleton and Newport, 2004), should be a natural consequence of winner-take-all production and learning from one's own utterances (Plan Reuse).

^6^In all Figure 1 studies, native speakers answered questions about cartoon pictures in which animate agents acted on animate or inanimate entities (e.g., a girl in a pool splashing a boy or a toy). Critical questions required speakers to describe the objects of actions; e.g., *What is green?* referred to the toy being splashed. Participants were not explicitly instructed to use relative clauses but frequently did so, with replies such as *The toy that the girl is splashing/that's being splashed {by the girl}*.

^7^A fuller treatment than is presented here would include the fact that object relatives with pronoun embedded subjects (*The boy/toy she splashed*….) have different production biases, different rates of production, and different comprehension patterns than the examples discussed here. We must also consider whether Easy First, Plan Reuse, and Reduce Interference provide an adequate account of why multiply-embedded object relatives, like Miller and Chomsky's (1963) *The rat [the cat [the dog chased] killed] ate the malt*, are essentially never produced, and the extent to which comprehension difficulty here can also be traced to ambiguity resolution gone awry rather than hard limits on working memory capacity.
